# Pellagra Found with Hookworm

**Published:** 1910-01

**Authors:** 


### Pellagra Found With Hookworm.

"Pellagra and hook worms travel hand in hand in their death-dealing work. Where the former is found there also may be found the latter, in many cases at least, boring its way to the vitals of the patient; and until the hook worm is routed, the successful treatment of pellagra is useless. To attempt to battle against pellagra, therefore, involves the turning of the artillery of the scientific world upon the hook worm."

This new development in the investigation of pellagra was brought out here in an able paper dealing with the disease, prepared by Dr. F. M. Sandwith, of London, Gresham Professor of Physics, and which was read before the International Conference on Pellagra by Dr. J. W. Babcock, Superintendent of the South Carolina Hospital for the Insane. More than 150 prominent physicians and scientists from all parts of the United States and representatives of foreign governments are in attendance.

